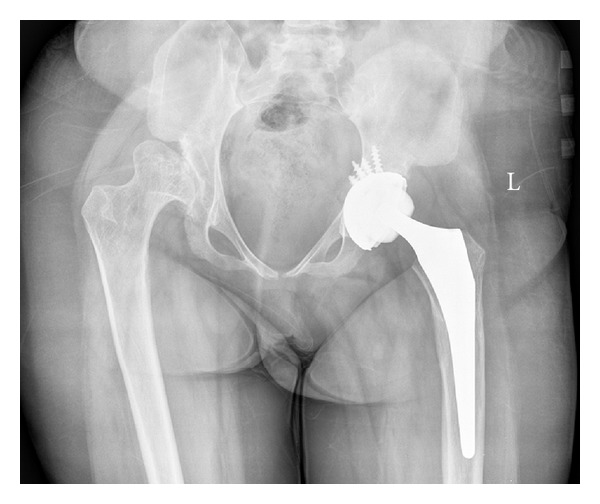# Total Hip Arthroplasty in Mucopolysaccharidosis Type IH

**DOI:** 10.1155/2011/832439

**Published:** 2012-01-26

**Authors:** S. O'hEireamhoin, T. Bayer, K. J. Mulhall

**Affiliations:** ^1^Department of Orthopaedic Surgery, Mater Misericordiae University Hospital, Eccles Street, Dublin 7, Ireland; ^2^Sports Surgery Clinic, Santry Demesne, Suite 4, Dublin 9, Ireland

## Abstract

Children affected by mucopolysaccharidosis (MPS) type IH (Hurler Syndrome), an autosomal recessive metabolic disorder, are known to experience a range of musculoskeletal manifestations including spinal abnormalities, hand abnormalities, generalised joint stiffness, genu valgum, and hip dysplasia and avascular necrosis. Enzyme therapy, in the form of bone marrow transplantation, significantly increases life expectancy but does not prevent the development of the associated musculoskeletal disorders. We present the case of a 23-year-old woman with a diagnosis of Hurler syndrome with a satisfactory result following uncemented total hip arthroplasty.

## 1. Introduction

Mucopolysaccharidosis type IH is an autosomal recessive lysosomal disorder. First described by Hurler in 1919 MPS IH has an incidence of 1 in 100,000 live births [1]. Hurler syndrome is due to a deficiency of *α*-L-iduronidase [2] resulting in inability to break down glycosaminoglycans and consequently infiltration of tissues with dermatan sulphate and heparin sulphate [3].

A variety of musculoskeletal abnormalities are associated with Hurler's syndrome. Spinal abnormalities include characteristic biconvex vertebral bodies, cervical myelopathy [4], odontoid hypoplasia, lumbar or thoracic kyphosis or hyperlordosis, and thoracolumbar cord compression [5]. Hip abnormalities include dysplasia, avascular necrosis (AVN), and failure of development of the superolateral femoral head [6]. Genu valgum, pronated feet, progressive clawing of the hands, and upper limb stiffness are also seen.

Treatment of Hurler syndrome with bone marrow transplantation, as proposed by Hobbs et al. [7], has resulted in increased life expectancy. Despite these advances progressive musculoskeletal disorders remain problematic [3, 6, 8].

## 2. Case Report

We present the case of a 23-year-old woman initially diagnosed on day 10 of life with mucopolysaccharidosis type IH (Hurler's syndrome).

She underwent bone marrow transplantation aged 20 months (1988). She had bilateral Salter's osteotomies for hip dysplasia aged five. She also had bilateral carpal tunnel release and known scoliosis and lumbar hyperlordosis.

She presented 4 years ago with gradually worsening thigh pain with ambulation. At that time, it was felt that her symptoms were spinal in origin and she had magnetic resonance imaging of her spine. This confirmed features typical of Hurler's, but while there was no evidence of spinal stenosis, there was moderate foraminal stenosis at L2/3, thought to be consistent with her clinical findings then. However, her symptoms progressed, and she became wheelchair bound and required regular daily analgesia secondary to progressive severe pain in her left groin and thigh. As a result of this progression she was referred for orthopaedic surgical assessment of both hips and spine.

**Figure 1:**
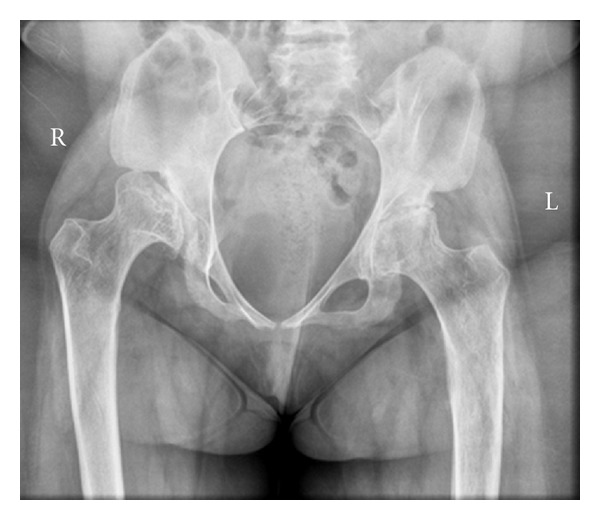


On presentation she was completely unable to weight bear and her pain was aggravated by movement and relieved by rest. Examination revealed significant hip irritability and restricted movement, with no evidence of neurological deficit or nerve root tension signs. Plain radiographs of her pelvis (Figure 1) revealed bilateral dysplasia and a right pseudo-acetabulum with moderately severe degeneration and femoral head collapse of the left hip.

After discussion with the patient and her family a decision was made to proceed with total hip arthroplasty.

Left THR was performed under spinal anaesthesia via the posterior approach. A “Trident” acetabular cup (Stryker, NJ, USA) and an “Accolade” femoral stem (Stryker), both uncemented, were inserted (Figure 2). An “X3” highly crosslinked ultrahigh weight polyethylene liner with 36 mm internal diameter was used. Her preexisting leg-length discrepancy due to dislocation of the contralateral hip was not corrected. It was felt that shortening the left side could have led to instability.

She recovered well postoperatively and at 2-year follow-up she had returned to her previous level of assisted community ambulation. Both she and her family expressed great satisfaction with her outcome.

## 3. Discussion

With increased life expectancy but continued musculoskeletal disorders orthopaedic management is likely to play an increasing role in the management of Hurler syndrome. This case illustrates the complexity of this multisystem disorder and the need for assessment of all possible causes of musculoskeletal pain in these patients before undertaking definitive management.

THR in this case resulted in resolution of symptoms and a satisfactory outcome. There is limited reporting of the use of THR in Hurler syndrome (MPS type I). Pryce Lewis and Gibson reported satisfactory results with THR in three patients suffering from MPS type III and IV [9]. In patients with Hurler syndrome and hip dysplasia Masterson et al. [10] have emphasised the role of hip containment procedure including various forms of innominate osteotomy; however, in advanced cases, total hip arthroplasty may play a role.

Although no long-term data is available on THR in these patients, it has been shown to be effective in the short-term for pain refractory to conservative measures and should be considered in these situations.

## Figures and Tables

**Figure 2:**